# Identification of Post-Transcriptional Modulators of Breast Cancer Transcription Factor Activity Using MINDy

**DOI:** 10.1371/journal.pone.0168770

**Published:** 2016-12-20

**Authors:** Thomas M. Campbell, Mauro A. A. Castro, Bruce A. J. Ponder, Kerstin B. Meyer

**Affiliations:** 1 Cancer Research UK Cambridge Institute, University of Cambridge, Li Ka Shing Centre, Robinson Way, Cambridge, United Kingdom; 2 Bioinformatics and Systems Biology Lab, Federal University of Paraná (UFPR), Polytechnic Center, Rua Alcides Vieira Arcoverde, Curitiba, PR, Brazil; University of South Alabama Mitchell Cancer Institute, UNITED STATES

## Abstract

We have recently identified transcription factors (TFs) that are key drivers of breast cancer risk. To better understand the pathways or sub-networks in which these TFs mediate their function we sought to identify upstream modulators of their activity. We applied the MINDy (Modulator Inference by Network Dynamics) algorithm to four TFs (ESR1, FOXA1, GATA3 and SPDEF) that are key drivers of estrogen receptor-positive (ER^+^) breast cancer risk, as well as cancer progression. Our computational analysis identified over 500 potential modulators. We assayed 189 of these and identified 55 genes with functional characteristics that were consistent with a role as TF modulators. In the future, the identified modulators may be tested as potential therapeutic targets, able to alter the activity of TFs that are critical in the development of breast cancer.

## Introduction

**Fig 1:**
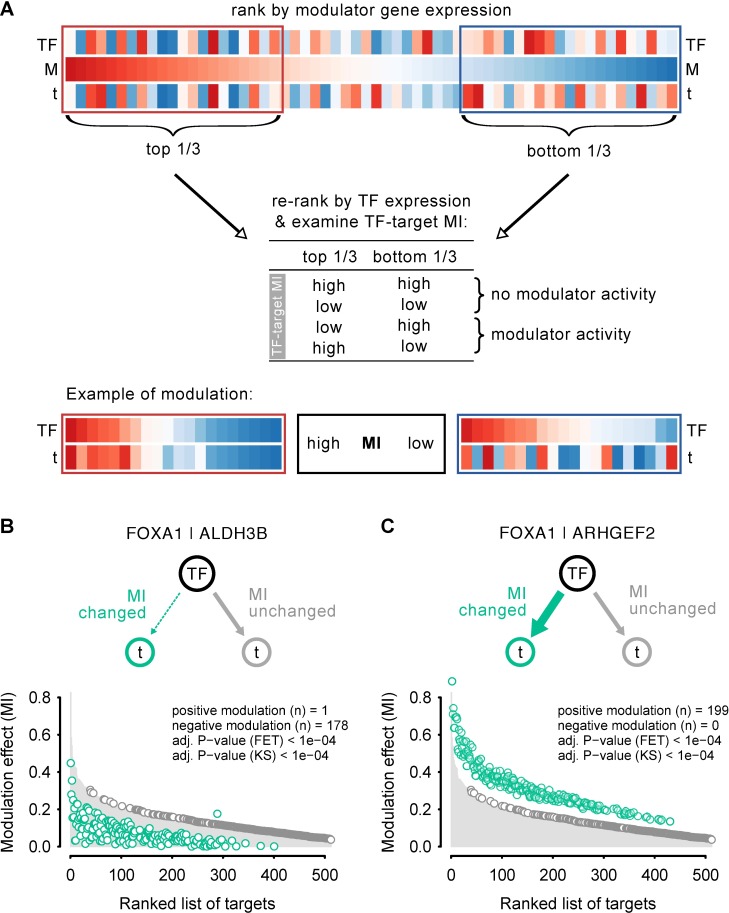
The MINDy algorithm. (A) Graphic representation of the MINDy analysis. For each tumour, gene expression activity is calculated for a given transcription factor (TF), a TF target (t) and a possible modulator (M), and represented as a vertical, coloured bar. Tumours are ranked by the expression value of the modulator. For the top 1/3 and bottom 1/3 of all tumours, samples are re-ranked by TF expression and mutual information (MI) between the TF and target is calculated. If similar MIs are obtained between the two groups no modulator activity is detected. However, if the MI value between the two groups is different, M has modulator activity. (B and C) Schematics and overall results for two predicted FOXA1 modulators, ALDH3B and ARHGEF2. The plots show the distribution of MI for the entire set of FOXA1 targets. The grey circles show the scaled MI values obtained when assessing MI for a TF-target interaction across all tumours. Targets are ranked, showing those with the highest mutual information on the left, those with the lowest towards the right. The green circles mark those targets for which a significant change in MI was observed when comparing the MI for TF|t for top 1/3 to the bottom 1/3 of all tumours ranked by the gene expression value for M (see materials and methods for calculations). ALDH3B (B) is an example of a modulator that reduces mutual information, ARHGEF2 (C) is a modulator able to increase TF|t similarities in expression. The inset text shows the resulting statistics that assess whether the number of modulated targets is different from what would be expected by chance, using two complementary tests: FET (Fisher’s Exact Test) and KS (Kolmogorov-Smirnov test). Of note, in the examples shown the TF-target pairs with the strongest MI show the greatest modulation, resulting a in a significant KS *P*-value.

Modulator Inference by Network Dynamics (MINDy) is a gene expression profile-based method to identify genes that modulate the transcriptional programme of a given transcription factor (TF). That is, MINDy is able to systematically identify genes that encode proteins that affect a TF’s activity without affecting its mRNA abundance. Modulators may act on the translation efficiency of the mRNA into protein, post-translationally modify the TF, affect the cellular localisation or turnover of the TF, form a transcriptional complex with the TF thereby changing its activity, or compete for its DNA binding site. The MINDy algorithm was introduced by the lab of Andrea Califano [1] and has been used to identify post-transcriptional modulators of TF activity in human B-cells [2]. Briefly, the MINDy algorithm interrogates a large gene expression profile dataset in order to identify candidate modulator genes able to alter the relationship between a TF and its regulon (set of target genes). For each TF of interest, a candidate modulator is tested by MINDy. Gene expression profiles from each of a set of samples (here, individual tumours) are ranked by the expression of the selected modulator, *M* (Fig 1). Sets of samples with high and low expression of the modulator are then selected (*M*-high and *M*-low). In each of the two sample sets, samples are then sorted according to TF expression and the extent of correlation in gene expression between the TF and its target genes is assessed. If the pattern of correlation differs significantly between *M*-high and *M*-low, then *M* is a modulator of the activity of that TF. The analysis also tests whether the modulator is a positive or negative one [1].

Here, we use the MINDy algorithm to identify modulators of the TFs ESR1, FOXA1, GATA3 and SPDEF (Fig 1). Each of these is an important driver of estrogen receptor-positive (ER^+^) breast cancer. In addition, these TFs are the master regulators (MRs) of the FGFR2 response, which is strongly associated with risk of breast cancer development [3,4]. ESR1, GATA3 and FOXA1 form part of the well-characterised estrogen receptor transcriptional network in ER^+^ breast cancer cells [5,6]. SPDEF is a novel co-regulator of the ESR1 transcriptional network. SPDEF is normally expressed in a range of epithelial cell types, especially in hormone-regulated tissues [7], and has been associated with cancer: SPDEF is overexpressed in breast cancer cells [8–10] but is often lost in high-grade, invasive tumours [11]. It was originally identified as a co-factor of the androgen receptor [12].

Having identified potential modulators of these TFs in ER^+^ breast cancer, we validate the MINDy findings with functional assays in order to demonstrate the biological relevance of our computational predictions.

## Materials and Methods

### MINDy algorithm

Modulators of transcription factor (TF) activity are assessed by conditional mutual information analysis as described elsewhere [1,2]. Briefly, this method takes a list of potential modulators and computes the conditional mutual information over the TF-target interactions of a given regulon. For each TF, the method measures the change in the mutual information between the TF and its targets conditioned to the gene expression of the modulator. The list of candidate modulators includes all genes annotated in the gene expression data, applying a modulator independence constraint to each test in order to exclude those candidates that are themselves correlated with the expression of the TF. The modulator inference was performed in *R* using the *tni*.*conditional* function in the RTN package (http://bioconductor.org/packages/RTN/) with 1000 permutations. The analysis pipeline has three main steps: (1) compute a regulatory network to derive regulons; (2) re-compute all regulons conditioned on the knowledge of a given candidate modulator. This is the MINDy algorithm, which tests whether the TF-target mutual information changes conditioned on the presence/absence of the modulator (it computes the differential mutual information). Here we also use a bootstrap analysis to check the stability of the inferred modulated targets, that is, we check the frequency that the inferred modulated targets can be observed in different subsamples; and (3) test whether the number of modulated targets is greater than would be expected by chance using FET (Fisher’s Exact Test) statistics. This step also tests the association between the observed modulated targets and the TF-target strength using KS (Kolmogorov-Smirnov) statistics, which aims to check whether the modulation happens in the strongest TF-target interactions. As a cut-off we chose an adjusted *P*-value of <0.001. To generate Fig 1, MI was calculated to generate grey dots. The shift from the original is calculated as dMI, which is scaled so that values are comparable across all targets:

$$dMI=\left(MI_{top}-MI_{bottom}\right)/\left(MI_{top}+MI_{bottom}\right)$$

The green circles represent the shift (MI_shift_) in the MI and values are calculated as:

$$MI_{shift}=MI_{original}+\left(MI_{original}*dMI\right)$$

### Cell culture

MCF-7 human breast cancer cells were cultured in DMEM (Invitrogen) supplemented with 10% FBS and antibiotics. T47D and ZR751 human breast cancer cells were cultured in RPMI (Invitrogen) supplemented with 10% FBS and antibiotics. HB2 mammary luminal epithelial cells were cultured in DMEM (Invitrogen) supplemented with 10% FBS, 5 μg/ml insulin, 1 μg/ml hydrocortisone and antibiotics. All cells were maintained at 37°C, 5% CO_2_. All cell lines were from the CRUK Cambridge Institute collection and cell line identity was confirmed by STR genotyping.

### Quantitative RT-PCR

1 μg of total RNA was reverse transcribed using the High Capacity cDNA Reverse Transcription Kit (Applied Biosystems) and qRT-PCR performed using cDNA obtained from 10 ng of total RNA. qRT-PCR was performed using an ABI 9800HT Sequence Detection System (Applied Biosystems) with SDS software version 2.3. All primers were specific for each gene of interest (Table 1). Amplification and detection were carried out in 384-well Optical Reaction Plates (Applied Biosystems) with Power SYBR Green Fast 2x qRT-PCR Mastermix (Applied Biosystems). All expression data were normalised to DGUOK expression. Primer-specificity was confirmed at the end of each qRT-PCR run through the generation of single peaks in melt-curve analysis. Data analysis was performed using the 2^-ΔΔCT^ method [13].

**Table 1:** Primers used in qRT-PCR to determine mRNA expression.

***Gene***	***Forward primer (5’-3’)***	***Reverse primer (5’-3’)***
*DGUOK*	GCTGGTGTTGGATGTCAATG	GCCTGAACTTCATGGTATTGG
*SPDEF*	CTGCTCAACATCACCGCAGATC	GGTGCTCTGTCCACAGGAGC
*FOXA1*	GGGGGTTTGTCTGGCATAGC	GCACTGGGGGAAAGGTTGTG
*FGFR2*	GTCAGTGAGAACAGTAACAACAAG	GTAGCCTCCAATGCGATGC
*CCND1*	AGAAGCTGTGCATCTACACCGACA	TGATCTGTTTGTTCTCCTCCGCCT

### Western immunoblotting

Cells were grown in 10 cm Petri dishes, washed in PBS and lysed on ice in RIPA buffer with cOmplete Mini EDTA-free protease inhibitor cocktail (Roche). Resulting cell lysates were passed through a fine-gauge syringe needle several times, centrifuged at 10,000 g for 1 minute and left at -80°C at least overnight. Protein samples were separated by SDS-PAGE using 4–12% Bis-Tris gels (Novex) for 2.5 hours (30 minutes at 60 V, 120 minutes at 120 V) and transferred by electrophoresis using an iBlot (Novex) for 7 minutes onto a nitrocellulose membrane (iBlot Gel Transfer Stacks; Novex). Successful transfer of protein was confirmed using Ponceau S Solution (Sigma). Membranes were “blocked” at room temperature for 1 hour with 5% (w/v) dried milk in Tris-buffered saline (TBS) with 0.1% Tween-20 (TTBS), washed 3x with TTBS and probed with the relevant primary antibody (anti-SPDEF, 1:200, Santa Cruz sc-67022; anti-FOXA1, 1:1000, Abcam ab5089; anti-FGFR2, 1:200, Santa Cruz sc-122; anti-β-actin, 1:5000, Cell Signalling) in blocking solution at 4°C overnight. Membranes were then re-washed with TTBS 3x and incubated with appropriate HRP-conjugated secondary antibody (anti-rabbit, 1:10,000, Amersham; anti-goat, 1:2000, Dako) in blocking solution at room temperature for 90 minutes. Following further washing with TTBS, blots were treated with SuperSignal West Chemiluminescent Substrate (Thermo Scientific) and immunoreactive proteins detected by exposure to film (FUJIFILM).

### Transient transfection of siRNA

Cells were transfected with ON-TARGETplus SMARTpool siRNA (Dharmacon) directed against *SPDEF* (L-020199-00), *FOXA1* (L-010319-00), *FGFR2* (L-003132-00), *CCND1* (L-003210-00), *MSL3* (L-012319-02), *ARHGEF2* (L-009883-00) and a control non-targeting pool (D-001810-01) using Lipofectamine RNAiMax Reagent (Invitrogen), according to manufacturer’s protocol. Following addition of the transfection complexes, cells were incubated at 37°C, 5% CO_2_ for 24–48 hours before experiments were performed.

### Transient transfection of siRNA for proliferation assays

Proliferation assays were performed in 96-well reverse transfection (RTF) plates (Dharmacon) and rehydration of siRNA was performed according to manufacturer’s protocol. Briefly, lyophilised siRNA for each of the 189 modulators of SPDEF and FOXA1, identified by MINDy, was rehydrated in the RTF plates using cell culture medium and Lipofectamine RNAiMax Reagent (Invitrogen), and siRNA transfection complexes were allowed to form. 4000 cells per well were then added to the plates, ready for proliferation analysis. The complete list of modulators against which siRNAs were targeted is available in S1 Table.

### Proliferation assay

Cells were plated at 4000 cells per well into 96-well plates and cell numbers monitored in real time by *in vitro* micro-imaging using an IncuCyte incubator (Essen BioScience), allowing for monitoring of cell proliferation by observing cell confluence. Images were taken every three hours and data consisted of an average of four separate images taken for each well. Assays were performed in triplicate on three separate occasions. Statistical analysis of proliferation data was performed using the **compareGrowthCurves** command in the *statmod* package in *R* (http://CRAN.R-project.org/package=statmod). Multiple testing correction was achieved using the Benjamini-Hochberg method.

### Molecular cloning

Three repeats of the SPDEF DNA binding consensus sequence was cloned into a pLUC2CP Luciferase Reporter Vector (kindly donated by the lab of David Neal, CRUK Cambridge Institute), following AscI/NheI digestion (AscI-(ACA GTG GTC CCG GAT TAT CGA)_3_-NheI), to generate a reporter construct in which luciferase gene expression is influenced by SPDEF protein binding. The orientation and sequence of the cloned plasmid was confirmed by DNA sequencing (GATC Biotech).

### Luciferase reporter assay

MCF-7 cells were plated at 0.5x10^5^ cells/well in 24-well dishes and left in complete medium until 50–70% confluent. If required, cells were first transfected with siRNA using Lipofectamine RNAiMax Reagent (Invitrogen), according to manufacturer’s protocol and left for 24 hours. Cells were then transfected with luciferase and β-galactosidase constructs together at a concentration of 0.5 and 0.1 μg per well, respectively, using FuGENE HD Transfection Reagent (Promega), according to manufacturer’s protocol. After 24 hours at 37°C, 5% CO_2_, cells were lysed with Reporter Lysis Buffer (Promega) and luciferase and β-galactosidase assays were performed on a PHERAstar FS Microplate Reader (BMG LABTECH) using the appropriate assay kits (Promega), according to manufacturer’s protocol. Transfection of each reporter construct was performed in triplicate in each assay and a total of three assays were performed on three separate days.

### Code availability

The source code developed in this study is publicly available from Bioconductor in the *R* package RTN (http://bioconductor.org/packages/RTN/).

## Results

### The MINDy algorithm identifies potential modulators of breast cancer risk TF activity

**Fig 2:**
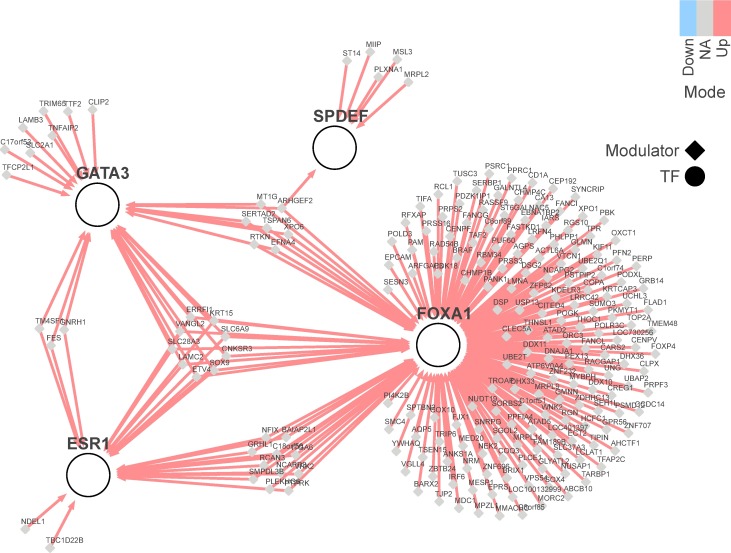
Post-transcriptional modulators of ESR1, GATA3, FOXA1 and SPDEF identified by MINDy. The MINDy algorithm was used to identify positive modulators, negative modulators and “in-modulators” of four TFs in ER^+^ breast cancer (ESR1, GATA3, FOXA1 and SPDEF). Just the positive modulators identified by MINDy are presented here. Negative modulators and in-modulators are presented in S1 and S2 Figs, respectively.

Previously, we have investigated TF involvement in modulating polygenic risk in breast cancer. We have identified 36 TFs whose regulons are significantly enriched for genes associated with breast cancer risk loci (termed “risk TFs”) [14], and four of these risk TFs (ESR1, FOXA1, GATA3 and SPDEF) have been identified as MRs of FGFR2-mediated risk in breast cancer [4]. In this study we applied the MINDy algorithm to ESR1, FOXA1, GATA3 and SPDEF, and their regulons. The MINDy algorithm (see materials and methods for details) was able to identify a total of 506 potential post-transcriptional modulators of ESR1, GATA3, FOXA1 and/or SPDEF activity. Of these 506 identified genes, 212 positively modulated one or several of the four TFs (Fig 2), 232 negatively modulated them (S1 Fig), and 62 lay in-between two or more of the TFs (we refer to these as the “in-modulators”), i.e. they were downstream of one of the TFs and upstream of another (S2 Fig). The vast majority of the positive and negative modulators that were identified affect the activity of FOXA1, with relatively few impacting on the activity of the other three TFs. We therefore chose to focus on FOXA1 for experimental validation of our results, and also SPDEF because it is a less well-studied, newly-identified ER^+^ breast cancer risk TF.

### Experimental validation and set-up

Previous studies [14] demonstrated that both FOXA1 and SPDEF are required for proliferation of ZR751 ER^+^ breast cancer cells. To determine the relevance of the identified modulators we therefore examined if downregulation of the positive modulators would affect cell proliferation in a FOXA1 or SPDEF-dependent manner. We focused our analysis on positive modulators since, in already rapidly proliferating cell lines, inhibition of growth might be more easily detectable than a potential increase in proliferation. Moreover, given the dependence of ER^+^ cell lines on FOXA1, positive modulators would be more attractive therapeutic targets.

**Fig 3:**
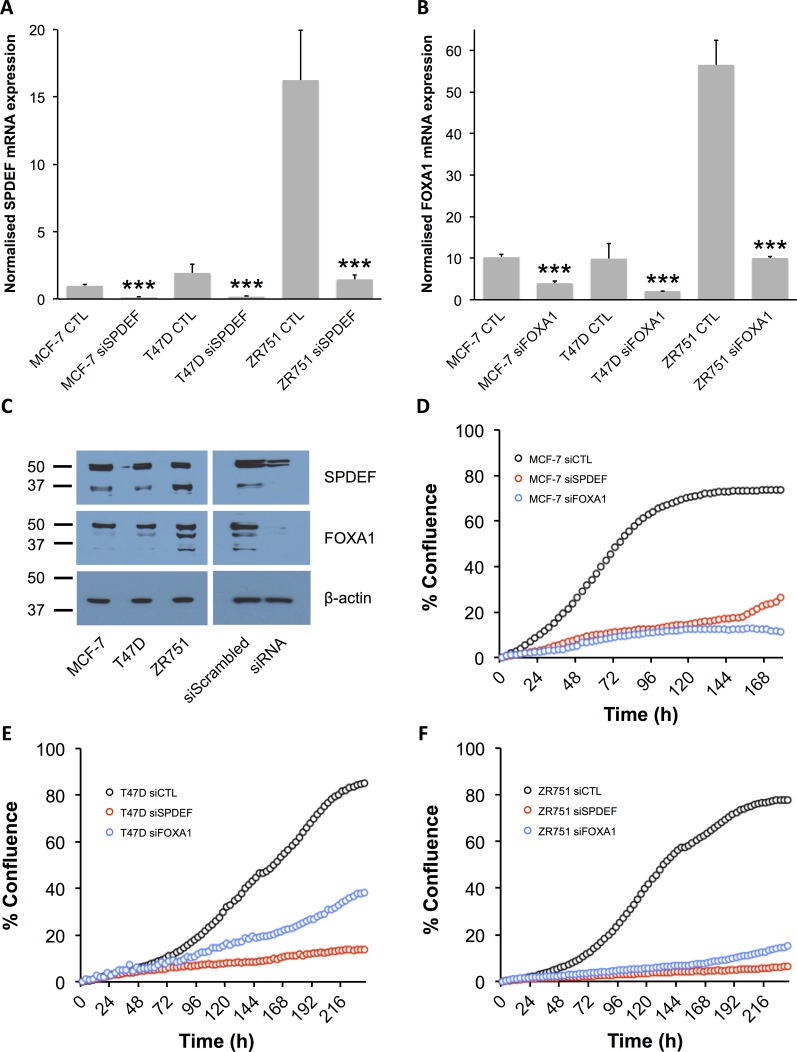
SPDEF and FOXA1 expression and activity in ER^+^ breast cancer cell lines. (A and B) Relative mRNA expression of SPDEF (A) and FOXA1 (B) in MCF-7, T47D and ZR751 ER^+^ breast cancer cells following transfection with siRNA directed against *SPDEF* and *FOXA1*, respectively. All data were normalised to DGUOK expression (*n* = 10, two separate experiments, *P*<0.001 (***), one-way ANOVA and SNK correction, error bars = SEM). (C) Representative Western immunoblots showing expression of SPDEF, FOXA1 and β-actin proteins in MCF-7, T47D and ZR751 ER^+^ human breast cancer cell lines (*n* = 3 for all blots). (D-F) Growth curves for MCF-7 (D), T47D (E) and ZR751 cells (F) following transfection with siRNA directed against *SPDEF* and *FOXA1*. Cells were transfected with scrambled, non-targeting siRNA as a control (siCTL).

**Fig 4:**
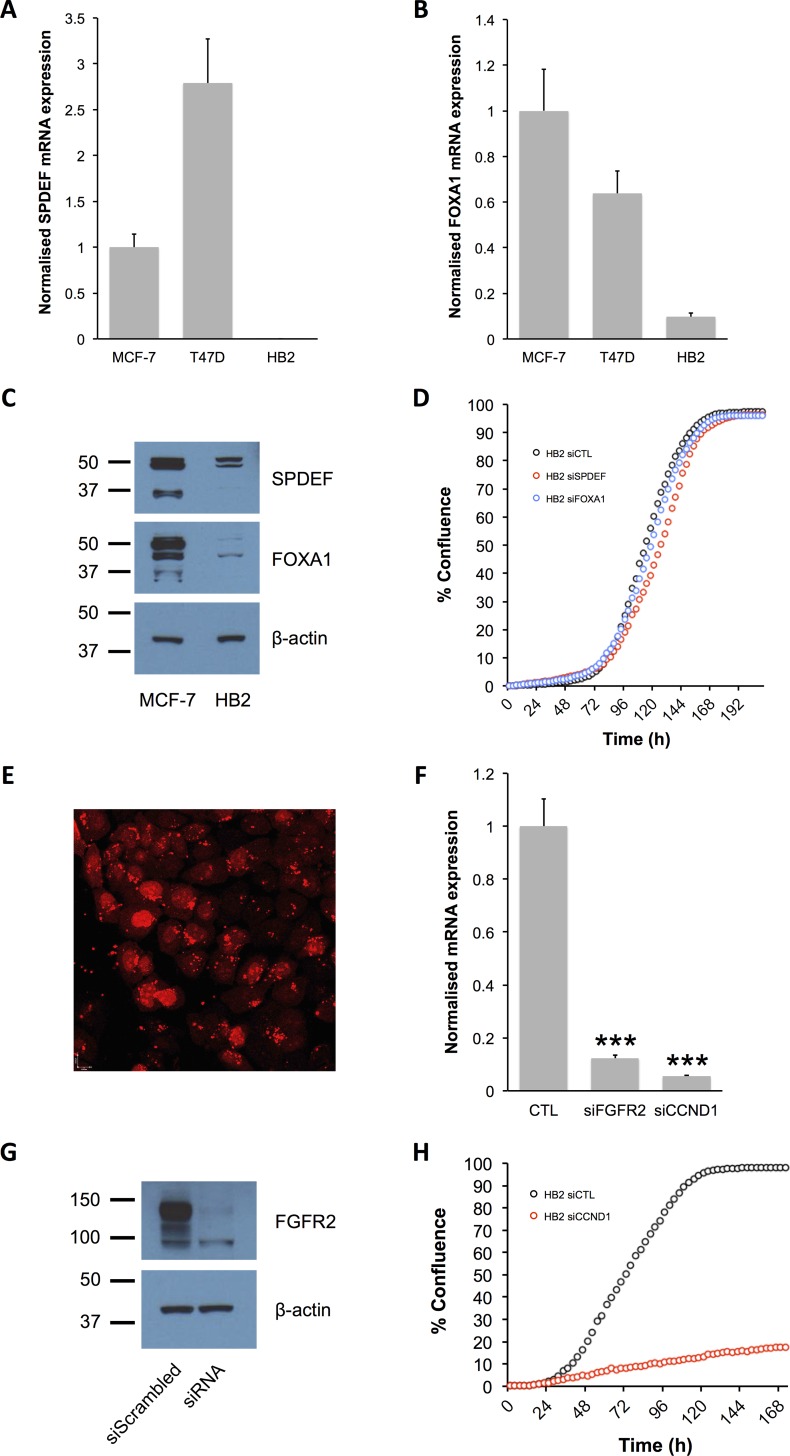
SPDEF and FOXA1 expression and activity in HB2 mammary epithelial cells. (A and B) Relative mRNA expression of SPDEF (A) and FOXA1 (B) in HB2 mammary epithelial cells. All data were normalised to DGUOK expression (*n* = 10, two separate experiments, error bars = SEM). (C) Representative Western immunoblots showing expression of SPDEF, FOXA1 and β-actin proteins in HB2 mammary epithelial cells (*n* = 3 for all blots). (D) Growth curves for HB2 cells following transfection with siRNA directed against *SPDEF* and *FOXA1*. Cells were transfected with scrambled, non-targeting siRNA as a control (siCTL). (E) Fluorescence image for HB2 cells transfected with siGLO Red Transfection Indicator using Lipofectamine RNAiMax Reagent. (F) Relative mRNA expression of FGFR2 and CCND1 in HB2 mammary epithelial cells following transfection with siRNA directed against *FGFR2* and *CCND1*, respectively. All data were normalised to DGUOK expression (*n* = 10, two separate experiments, *P*<0.001 (***), one-way ANOVA and SNK correction, error bars = SEM). (G) Representative Western immunoblots showing expression of FGFR2 and β-actin proteins in HB2 mammary epithelial cells following transfection with siRNA directed against *FGFR2* (*n* = 3 for all blots). (H) Growth curves for HB2 cells following transfection with siRNA directed against *CCND1*. Cells were transfected with scrambled, non-targeting siRNA as a control (siCTL).

We first characterised SPDEF and FOXA1 expression and activity in three ER^+^ breast cancer cell lines (MCF-7, T47D and ZR751). Both SPDEF and FOXA1 mRNA and protein were expressed in all three ER^+^ breast cancer cell lines, and their expression levels were significantly reduced following transfection of siRNA against the TFs (Fig 3A–3C). In all three ER^+^ cell lines, transfection of siRNA against SPDEF or FOXA1 also significantly reduced cell proliferation compared with transfection of a control, non-targeting siRNA sequence (Fig 3D–3F). This suggests that proliferation of the ER^+^ breast cancer cell lines was dependent on SPDEF and FOXA1 activity. In contrast, the HB2 cell line is a human mammary epithelial cell line with hardly detectable levels of SPDEF and very low levels of FOXA1 compared with ER^+^ breast cancer cell lines (Fig 4A–4C), whose growth does not depend on SPDEF and FOXA1 activity (Fig 4D). We therefore used HB2 cells as controls to establish whether the activity of the identified modulator was FOXA1 or SPDEF-dependent or occurred via a non-specific mechanism. In order to satisfy ourselves that siRNA is able to get into the HB2 cells, transfection experiments were performed. siRNA transfection in HB2 cells was efficient, as demonstrated by transfection of siGLO RED Transfection Indicator (Fig 4E). Also, transfection of siRNAs directed against *FGFR2* and *CCND1* significantly reduced levels of mRNA for these genes (Fig 4F) and reduced levels of FGFR2 protein (Fig 4G). Furthermore, HB2 cell proliferation was inhibited following transfection of siRNA directed against *CCND1*, unlike with siRNA directed against *SPDEF* and *FOXA1* (Fig 4H).

The following criteria were used to select a list of modulators to validate. Firstly, only positive modulators were chosen (Fig 2). Secondly, all positive modulators of SPDEF that were identified by MINDy were included (6 modulators). Thirdly, all of the in-modulators (S2 Fig) that positively regulated SPDEF (3 modulators) were included in the analysis. Finally, the long list of positive FOXA1 modulators was ranked by *P*-value and a cut-off of 0.001 was employed, with consensus between the two cohorts. This gave a total of 189 positive post-transcriptional modulators of SPDEF and FOXA1 to be tested (S2 Table; 6 positive modulators of SPDEF, including one that is also a positive modulator of FOXA1 and GATA3, 180 positive modulators of FOXA1, including some that are also positive modulators of ESR1 and/or GATA3, and 3 in-modulators that positively regulate SPDEF).

### MINDy-identified modulators of breast cancer risk TF activity affect ER^+^ breast cancer cell proliferation

**Fig 5:**
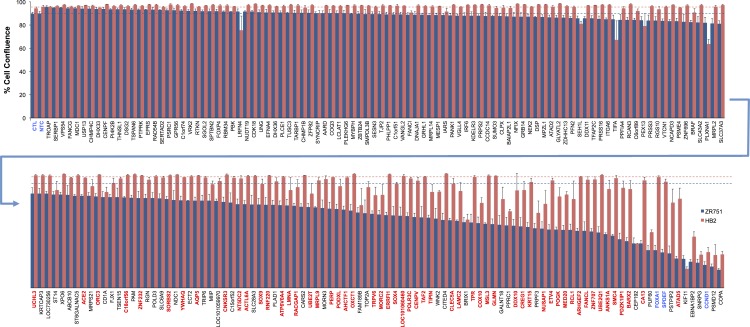
Experimental validation of potential SPEDF and FOXA1 modulators. Proliferation of ZR751 and HB2 cells was assayed following transfection with siRNA directed against modulators of SPDEF and FOXA1, identified by MINDy analysis. ZR751 cells are dependent on SPDEF and FOXA1 for proliferation whereas HB2 cells do not require these TFs for growth. The plot shows cell proliferation, represented as % cell confluence at the time-point when cell confluence plateaus in the non-targeting siRNA control treatment (NTC), measured using an IncuCyte imaging system (only a single time-point is represented in the plot). The proliferation screen, which was statistically analysed using the **compareGrowthCurves** command in the *statmod* package in *R* (http://CRAN.R-project.org/package=statmod), with multiple testing correction carried out using the Benjamini-Hochberg method (S3 Table, S3 Fig), identified a number of modulators that have a consistent cell type-specific effect (55 out of 189 modulators), and these are highlighted in red on the x-axis. Control treatments are highlighted in blue on the x-axis. The dashed lines show maximum percent cell confluence achieved with the NTC treatment for ZR751 and HB2 cells. CTL: untransfected control; NTC: non-targeting siRNA control.

To test the activity of these 189 modulators, ZR751 (highest expression of SPDEF and FOXA1; Fig 3) and HB2 cells were transfected with siRNAs targeting these modulators and cell proliferation was assayed. Statistical analysis of proliferation data was performed using the **compareGrowthCurves** command in the *statmod* package in *R* (http://CRAN.R-project.org/package=statmod), with multiple testing correction carried out using the Benjamini-Hochberg method. Using this statistical approach, we found that 55 modulators significantly inhibited growth of the ZR751 cell line without affecting proliferation of the HB2 cell line (Fig 5, S3 Table, S3 Fig). In this regard, these 55 modulators behave like the TFs they are positively modulating, in that their reduced expression/activity results in a perturbed proliferative response, which is cell type-specific. Our positive controls, SPDEF and FOXA1, are towards the far right of the plot displayed in Fig 5, confirming that knocking down expression of the TFs has a large effect on ZR751 cell proliferation compared with HB2 cell proliferation. Knocking down expression of 14 of the 189 modulators inhibited growth of both cell lines, presumably as a result of off-target effects not associated with SPDEF/FOXA1 modulation. For example, siRNA directed against *CCND1* (encodes cyclin D1, an important cell cycle protein), was used as a control in the assay as knocking down its expression should reduce proliferation in all cell lines, not just those dependent on SPDEF and FOXA1 activity. Any modulator which showed a response pattern similar to *CCND1* in the assay was disregarded. Overall, we found that 53 of the FOXA1 modulators had an effect on proliferation and two SPDEF modulators, MLS3 and ARHGEF2, significantly reduced proliferation specifically in ZR751 cells.

### Validated modulators are frequently mutated in breast cancer

**Fig 6:**
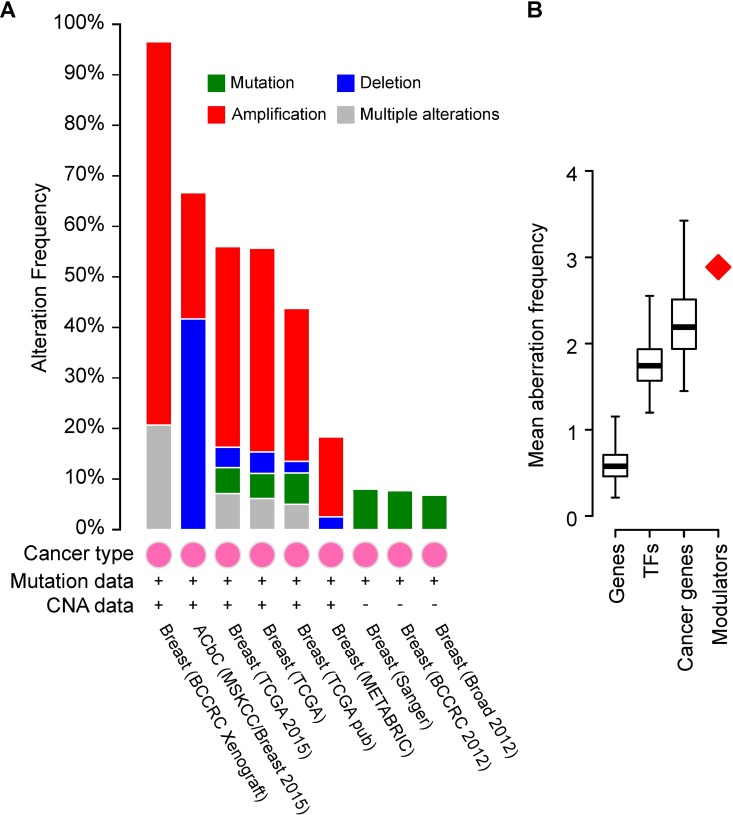
The MINDy-identified modulators of SPDEF and FOXA1 are frequently altered in breast cancer. (A) Plot from cBioPortal for Cancer Genomics showing the alteration frequency of the 55 SPDEF and FOXA1 modulators validated in this study, from nine different breast cancer studies. (B) Mean aberration frequency (mutations or copy number alterations) of the 55 SPDEF and FOXA1 modulators in comparison to sets of 55 random genes (empirical *P*<0.001; box-plot whiskers extend to the 1^st^ and 99^th^ percentiles of the random distribution with 10,000 random sets). Aberration frequencies for sets of random TFs and cancer genes are also shown.

To obtain orthogonal data for the importance of the identified modulators we examined whether the genes of the 55 validated modulators are altered in cancer. Studies with available copy number alteration (CNA) data show that amplification of the modulator genes is very common in breast cancer [15–17] (Fig 6A). Moreover, mutations in these genes are present in up to 10% of the samples tested in the available studies [15–20]. Collectively, our 55 modulators have a significantly increased frequency of alterations in comparison to both random genes and annotated cancer-related genes for which mutations have been causally implicated in cancer [21] (Fig 6B). These data support the functional importance of the identified modulators.

### MINDy-identified modulators of SPDEF activity affect SPDEF-driven transcription

**Fig 7:**
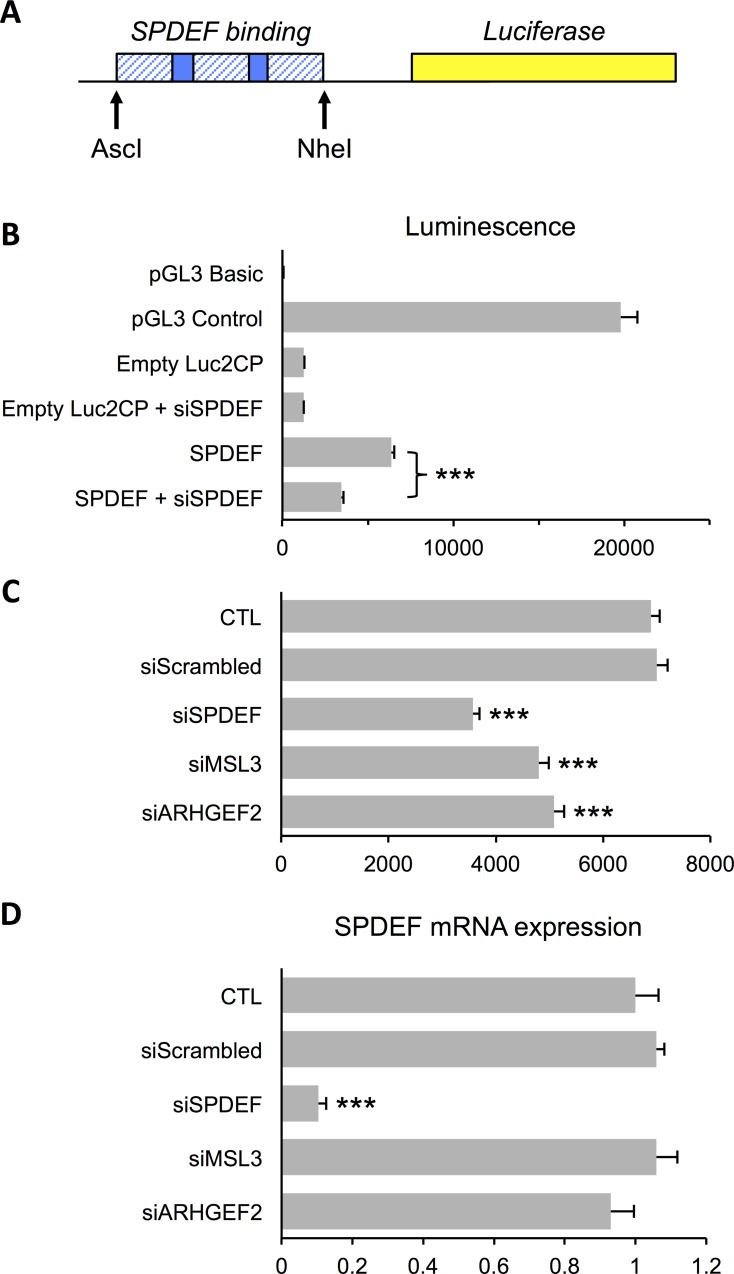
MINDy-identified modulators of SPDEF activity affect SPDEF-driven transcription. (A) Schematic depiction of the reporter construct used in the luciferase reporter assays. (B) Luciferase luminescence in MCF-7 cells 24 hours post-transfection of reporter construct containing SPDEF binding domains (SPDEF), normalised to β-galactosidase expression (*n* = 9, three separate experiments, *P*<0.001 (***), one-way ANOVA and SNK correction, error bars = SEM). pGL3 Basic/pGL3 Control: control luciferase reporter vectors; Empty Luc2CP: Empty vector control; siSPDEF: SPDEF siRNA treatment. (C) Luciferase luminescence 24 hours post-transfection of reporter construct containing SPDEF binding domains into MCF-7 cells treated with siRNA directed against *SPDEF*, *MSL3* and *ARHGEF2*, normalised to β-galactosidase expression (*n* = 9, three separate experiments, *P*<0.001 (***), one-way ANOVA and SNK correction, error bars = SEM). CTL: untransfected control; siScrambled: non-targeting siRNA control. (D) Relative mRNA expression of SPDEF in MCF-7 cells following transfection with siRNA directed against *SPDEF*, *MSL3* and *ARHGEF2*. All data were normalised to DGUOK expression (*n* = 10, two separate experiments, *P*<0.001 (***), one-way ANOVA and SNK correction, error bars = SEM) CTL: untransfected control; siScrambled: non-targeting siRNA control.

As outlined in the introduction, modulators identified by MINDy may post-transcriptionally and/or post-translationally modify the TF, affect its cellular localisation or turnover, form a transcriptional complex with the TF, or compete for its DNA binding site. Ultimately, the modulator must alter the ability of the TF to drive transcription. We tested this on the two SPDEF modulators that affected ZR751 cell proliferation (Fig 5). Transcriptional activity was assayed by generating SPDEF-driven luciferase reporter constructs (Fig 7A) to ask if removal of the MINDy-identified modulators of SPDEF would alter the luciferase read-out. As a positive control in our assay we transfected the SPDEF-luciferase reporter construct into ZR751 cells and observed that luciferase luminescence was reduced by 46% when siRNA directed against *SPDEF* was co-transfected (Fig 7B). Similarly, when siRNA directed against *MSL3* and *ARHGEF2*, two validated modulators of SPDEF, was transfected into ZR751 cells, luciferase luminescence was reduced by 30% and 26%, respectively (Fig 7C), without having any affect on SPDEF expression levels (Fig 7D). These two SPDEF modulators were identified by MINDy, and the proliferation screen (Fig 5) suggests that they are required by ZR751 cells for growth. These data therefore show that MINDy is able to identify biologically-relevant modulators of TFs that can alter the TF’s transactivational activity.

## Discussion

Here we demonstrate that the MINDy algorithm is able to identify post-transcriptional modulators of key TFs of ER^+^ breast cancer. We have found and validated novel modulators of FOXA1 and SPDEF, both of which are of special interest in breast cancer. FOXA1 may be a particularly relevant therapeutic target since cancers that have become resistant to hormone treatment may still be dependent on FOXA1 for proliferation [22]. Also, SPDEF may be an important regulator of cell metastasis, not just in prostate but also in breast cell lines [23]. Both TFs also affect the risk of developing breast cancer [4] and are therefore of interest in designing preventative strategies.

To seek experimental validation of the candidate modulators, we transfected siRNA libraries directed against positive modulators into SPDEF and FOXA1-dependent and -independent cell lines, and potential biologically-relevant modulators were identified on the basis of affecting proliferation in one, but not the other cell line. Of the 189 modulators selected for study, over a quarter (55) were shown to affect ER^+^ breast cancer cell growth when compared to a cell line that does not require the TFs in order to proliferate. The primary function of 25 out of these 55 modulators is classed as having a role in either cell cycle or transcription (GO term analysis), supporting the idea that these modulators might affect the activity of a TF required for cell proliferation. Validation assays based on other cellular phenotypes or specific protein function may be able to validate additional modulators identified in our computational analysis.

As yet, we do not understand why MINDy was able to identify such a large number of modulators of FOXA1, while only finding relatively few factors that affected ESR1, given that these two TFs share many binding sites and target genes [22,24,25]. One possible explanation may lie in the fact that ESR1 activity depends on the presence of estrogen, which may not be universally available within the tumour. The link between gene expression and target genes may therefore be less strong and MINDy less able to find modulators. There may be other confounding factors. Regulatory processes associated with the cell cycle are largely modulated by protein phosphorylation cascades. Such alterations, potentially leading to protein degradation, may break the correlation of gene expression between the TF and its targets that the MINDy algorithm relies on.

In conclusion, our analysis of the effect of potential modulators of SPDEF and FOXA1 activity in ER^+^ breast cancer suggests that MINDy and other such computational tools have the power to identify valid modulators of TF activity that warrants further follow-up work. For SPDEF we found two modulators which have the ability to reduce SPDEF-driven transcription. MLS3 is a component of the MLS complex responsible for the majority of histone H4 acetylation at Lys16. GO annotations for this nuclear protein include chromatin organisation and transcription. ARHGEF2 is a primarily cytoplasmic Rho/Rac guanine nucleotide exchange factor involved in signalling by G-protein coupled receptors. Further functional follow-up analysis of these proteins in the context of ER^+^ breast cancer may be warranted. Also, a large number of FOXA1 modulators that can affect proliferation were found and future studies will have to assess their contribution to driving the cancer phenotype.

TFs are notoriously difficult targets to drug [26]. Therefore, the availability of tools that can accurately identify modulators of TFs, which could prove to be more tractable therapeutic targets, is very appealing.

## Supporting Information

S1 Fig Negative modulators of ESR1, GATA3, FOXA1 and SPDEF, identified by MINDy. (PDF)

S2 Fig “In-modulators” of ESR1, GATA3, FOXA1 and SPDEF, identified by MINDy. The in-modulators refer to those modulators that lie in-between two or more of the TFs of interest, i.e. their activity is modulated by one of the TFs and they modulate the activity of another of the TFs.
(PDF)

S3 Fig Example growth curves. 12 of the 69 significant (*P*-value = 0.005) modulator gene example growth curves obtained in ZR751 breast cancer cells using the growth curve analysis are shown. Red: test gene siRNA; Green: non-targeting control siRNA. Statistical analysis of proliferation data was performed using the **compareGrowthCurves** command in the *statmod* package in *R* (http://CRAN.R-project.org/package=statmod). Multiple testing correction was achieved using the Benjamini-Hochberg method.
(PDF)

S1 Table siRNA plate layout. (XLS)

S2 Table Modulator list. (XLSX)

S3 Table MINDy-identified modulator genes showing statistically significant impact on proliferation of ZR751 cells compared to control. (DOCX)
