# Supplementary figures and images for: Identification of Post-Transcriptional Modulators of Breast Cancer Transcription Factor Activity Using MINDy
Source: PLoS One. 2016 Dec 20;11(12):e0168770. doi: 10.1371/journal.pone.0168770 (PMC5173250; doi:10.1371/journal.pone.0168770)

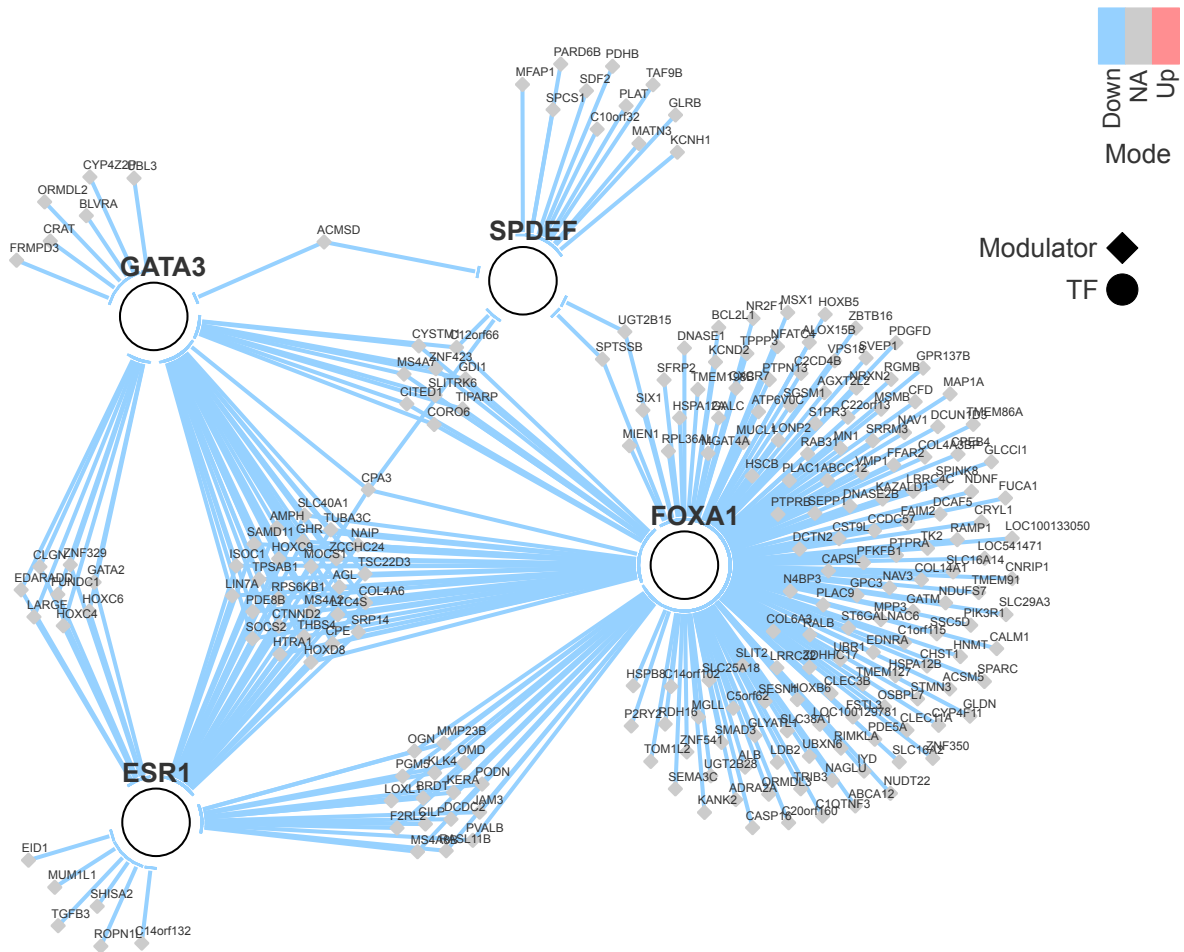

Supplement: S1 Fig — (PDF) [file pone.0168770.s001.pdf]

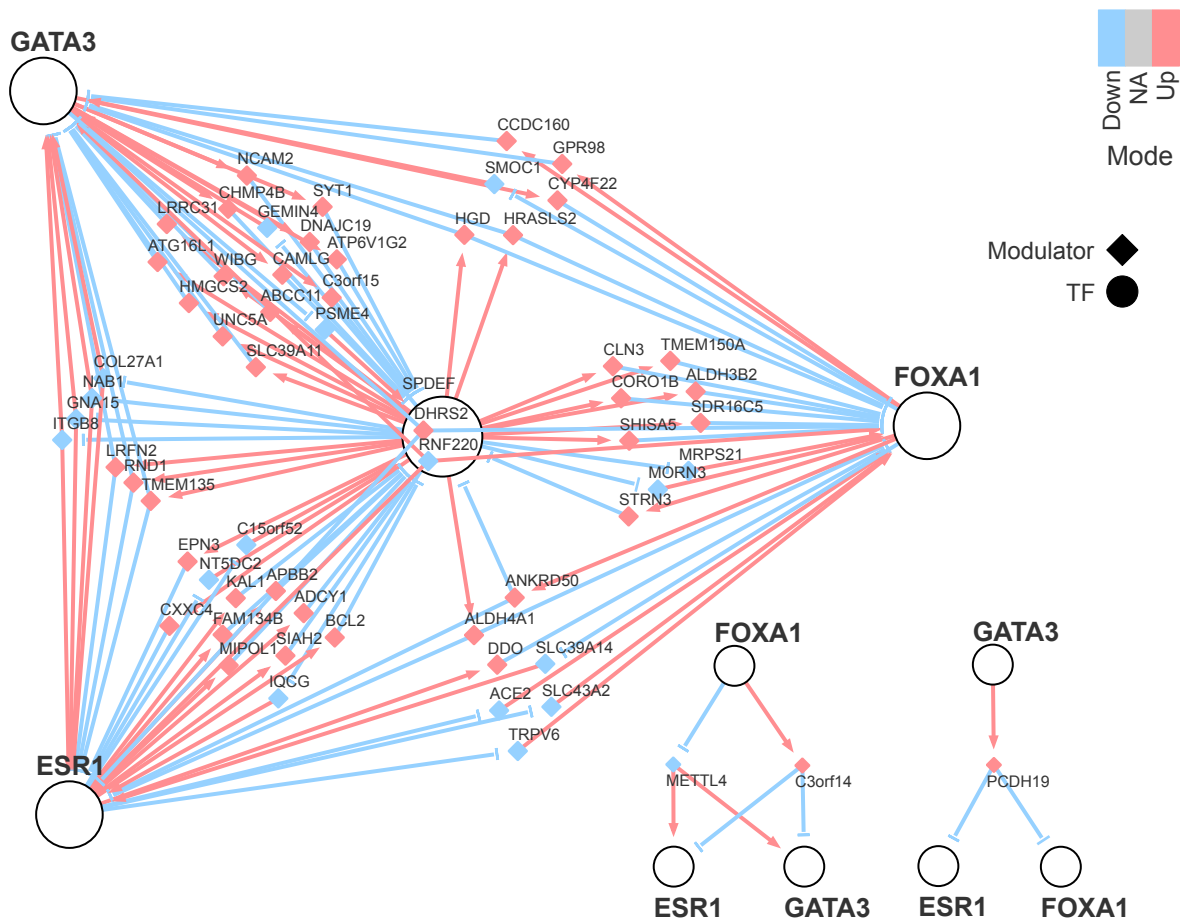

Supplement: S2 Fig — The in-modulators refer to those modulators that lie in-between two or more of the TFs of interest, i.e. their activity is modulated by one of the TFs and they modulate the activity of another of the TFs. (PDF) [file pone.0168770.s002.pdf]

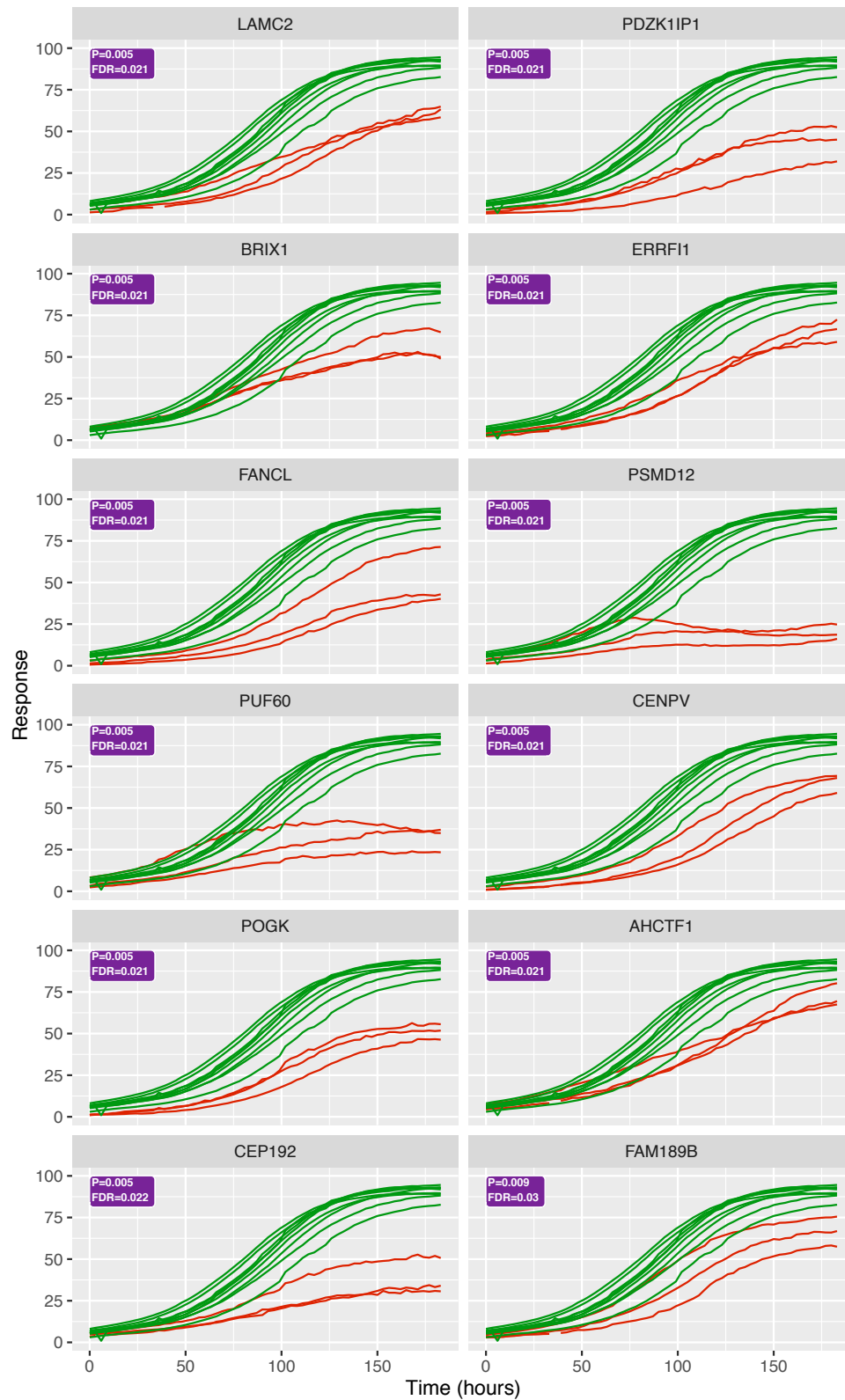

Supplement: S3 Fig — 12 of the 69 significant (P-value = 0.005) modulator gene example growth curves obtained in ZR751 breast cancer cells using the growth curve analysis are shown. Red: test gene siRNA; Green: non-targeting control siRNA. Statistical analysis of proliferation data was performed using the compareGrowthCurves command in the statmod package in R (http://CRAN.R-project.org/package=statmod). Multiple testing correction was achieved using the Benjamini-Hochberg method. (PDF) [file pone.0168770.s003.pdf]
