# Supplementary material for: Identification of Post-Transcriptional Modulators of Breast Cancer Transcription Factor Activity Using MINDy
Source: PLoS One. 2016 Dec 20;11(12):e0168770. doi: 10.1371/journal.pone.0168770 (PMC5173250; doi:10.1371/journal.pone.0168770)
Supplement: S3 Table — (DOCX) [file pone.0168770.s006.docx]

| *Modulator gene* | *t statistic* | *P-value* | *FDR* | *Possible non-specific* |
| --- | --- | --- | --- | --- |
| *ACE2* | 2.57 | 0.014 | 0.040 |  |
| *ACTL6A* | 3.01 | 0.009 | 0.030 |  |
| *AHCTF1* | 3.76 | 0.005 | 0.021 |  |
| *ANKS1A* | 8.20 | 0.004 | 0.021 |  |
| *AQP5* | 2.92 | 0.014 | 0.040 |  |
| *ARHGEF2* | 7.57 | 0.004 | 0.021 |  |
| *ATAD5* | 10.21 | 0.005 | 0.021 |  |
| *ATP6V0A4* | 4.57 | 0.004 | 0.021 |  |
| *BARX2* | 7.93 | 0.004 | 0.021 |  |
| *BRIX1* | 3.79 | 0.005 | 0.021 | Yes |
| *C18orf56* | 2.40 | 0.018 | 0.049 |  |
| *CA13* | 8.69 | 0.004 | 0.021 |  |
| *CENPV* | 5.95 | 0.005 | 0.021 |  |
| *CEP192* | 6.14 | 0.005 | 0.022 | Yes |
| *CLEC5A* | 5.02 | 0.004 | 0.021 |  |
| *CNKSR3* | 2.75 | 0.014 | 0.040 |  |
| *COPA* | 11.25 | 0.005 | 0.021 | Yes |
| *COX10* | 4.44 | 0.004 | 0.021 |  |
| *CREG1* | 5.46 | 0.004 | 0.021 |  |
| *DDX10* | 3.61 | 0.009 | 0.030 |  |
| *EBNA1BP2* | 8.63 | 0.005 | 0.021 | Yes |
| *ERRFI1* | 5.41 | 0.005 | 0.021 |  |
| *ETV4* | 5.27 | 0.005 | 0.021 |  |
| *FAM189B* | 3.95 | 0.009 | 0.030 | Yes |
| *FANCL* | 5.78 | 0.005 | 0.021 |  |
| *GALNT18* | 4.79 | 0.004 | 0.021 | Yes |
| *GLMN* | 6.78 | 0.004 | 0.021 |  |
| *KIF11* | 9.06 | 0.004 | 0.021 | Yes |
| *KRT15* | 5.74 | 0.005 | 0.021 |  |
| *LAMC2* | 6.01 | 0.005 | 0.021 |  |
| *LMNA* | 4.05 | 0.009 | 0.030 |  |
| *LOC101060460* | 3.86 | 0.009 | 0.030 |  |
| *MED20* | 4.64 | 0.004 | 0.021 |  |
| *MORC2* | 5.41 | 0.005 | 0.021 |  |
| *MRPL9* | 2.78 | 0.009 | 0.030 |  |
| *MSL3* | 4.96 | 0.005 | 0.021 |  |
| *NT5DC2* | 3.20 | 0.013 | 0.040 |  |
| *NUSAP1* | 4.32 | 0.004 | 0.021 |  |
| *ORC3* | 3.12 | 0.013 | 0.040 |  |
| *OXCT1* | 4.40 | 0.004 | 0.021 |  |
| *PDZK1IP1* | 6.82 | 0.005 | 0.021 |  |
| *PERP* | 4.89 | 0.005 | 0.021 |  |
| *PODXL* | 5.42 | 0.005 | 0.021 |  |
| *POGK* | 7.38 | 0.005 | 0.021 |  |
| *POLR3C* | 5.82 | 0.004 | 0.021 |  |
| *PPRC1* | 6.69 | 0.004 | 0.021 | Yes |
| *PRPF3* | 6.89 | 0.005 | 0.021 | Yes |
| *PSMD12* | 9.34 | 0.005 | 0.021 | Yes |
| *PSTPIP2* | 7.09 | 0.005 | 0.021 | Yes |
| *PUF60* | 6.26 | 0.005 | 0.021 | Yes |
| *RACGAP1* | 2.98 | 0.010 | 0.030 |  |
| *RCL1* | 5.45 | 0.005 | 0.021 |  |
| *RNF220* | 2.77 | 0.018 | 0.050 |  |
| *SMC4* | 6.18 | 0.005 | 0.021 |  |
| *SNRPG* | 9.37 | 0.004 | 0.021 | Yes |
| *SORBS2* | 4.50 | 0.005 | 0.021 |  |
| *SOX4* | 4.20 | 0.009 | 0.030 |  |
| *SOX9* | 4.14 | 0.004 | 0.021 |  |
| *TAF2* | 3.24 | 0.009 | 0.030 |  |
| *TIPIN* | 4.05 | 0.005 | 0.021 |  |
| *TPR* | 3.98 | 0.009 | 0.030 |  |
| *TRPV6* | 3.83 | 0.009 | 0.030 |  |
| *UBE2Q1* | 6.50 | 0.004 | 0.021 |  |
| *UBE2T* | 4.16 | 0.009 | 0.030 |  |
| *UCHL3* | 2.83 | 0.014 | 0.040 |  |
| *WNK2* | 3.96 | 0.009 | 0.030 | Yes |
| *YWHAQ* | 3.65 | 0.009 | 0.030 |  |
| *ZNF232* | 3.06 | 0.009 | 0.030 |  |
| *ZNF707* | 7.32 | 0.005 | 0.021 |  |
| Data generated from proliferation assays following siRNA knockdown of modulator genes, using the compareGrowthCurves command in the *statmod* package in *R* (<http://CRAN.R-project.org/package=statmod>), with multiple testing correction carried out using the Benjamini-Hochberg method.  The column “Possible non-specific” indicates genes that had a *P*-value<0.05 for HB2 cells. | | | | |
